# Heredity and Health

**Published:** 1918-11-23

**Authors:** 


### HEREDITY AND HEALTH.

(FROM A CORRESPONDENT.)

THE amount of consideration devoted to questions of health and population is phenomenal and portentous. The short of memory think it only a result of the war, and that it has sprung from recognition of the great loss of human lives the war has caused. But for years before the war began there was national uneasiness, and for years before the crowd turned attention to the matter thoughtful men had been studying the question with ever-increasing anxiety, even with consternation. To-day we are holding Baby Weeks and passing Maternity Bills and agitating for a Ministry of Health and making other sporadic and futile exhibitions of activity; but no statesman has appeared with the vision to discern the cause of this questioning, nor the wisdom to propose far-reaching remedies; no prophet has clearly cried the evil nor shed light.

What is the evil? The evil is that of every hundred men and women in England an immense percentage is physically feeble, unpleasingly formed in natural parts, always ill. Of every other species that the Author of the Universe has caused to be evolved upon the earth the great majority is physically excellent, happy, and in the enjoyment of perfect health. Consider the birds of the air and the creatures of moorland and mountain, sea and desert. Only of Man, Man who flatters his kind that it is the greatest, most glorious, and final creation of the Almighty Maker, may it be said that a large part is miserable, frequently ill, always out of health, and far below the most excellent in beauty of form and feature, in capacity for individual existence, and in power to do a fair share for the community.

Commonplace things are revelations of the universe. The observation of them is the way to Truth. He who will watch the common things around him will be struck by the slight divergence from the average, the smallness of the difference between the best and the worst, of the individuals of any species of living thing, living in the hand of the Almighty, no man interfering with the way of life; and by the vastness of the difference between the finest of mankind and the poorest specimens. As he ponders he will remember that there is the same great gap between the best and the worst of the animals under the hand of man and moulded into shape by the exercise of the will, the reason, and the power to plan that is given to Man in so much greater measure than to other creatures. Vast is the difference between the magnificent stallion that shall sire great winners and the weedy screw condemned to the cab-rank; between the pedigree bull and a crofter's scrubby cattle. It may occur to him that this ill-health, this misery, this existent great inferiority of the worst to the best may be the result of a mistake of Man and not a decree of the Origin of the Universe, may be the result of wrong use of that greater power to reason evolved in Man and by virtue of which Man fashions environment. If he decide it is so, he may try to seek out the mistake, and succeed or fail. In either event he will have had the joy of endeavour.

He may premise physical feebleness, chronic ill-health, want of capacity to obtain a living are results of environment; an environment of slums and miserable dwellings, insanitary towns and overcrowding. If so, it was Man made the slums, built the miserable dwellings, refused to cleanse the towns. But just in those countries where the ill-health is of the highest, the physical feebleness most marked, and the necessity for assistance to survive greatest, the towns are greatly cared for, the most of the dwellings well built, the slum areas but a small portion. And in lands where squalid hovels house the population, and every town is an insanitary slum, often physical feebleness is no more marked and the strength of the feebler half of the population greater than the strength of the feebler half of dwellers in more enlightened lands, and the average health of the people, in the absence of acute infections, of higher standard than is enjoyed by the average of those that dwell under a more elaborate civilisation. Nor are physical feebleness, chronic ill-health, and want of capacity absent from the population that has most favourable environment. On the contrary, all are exceedingly common amongst it. Men and women slight of bone, thin of chest, light of muscle, nervous, dyspeptic, always calling in the doctor, are common in wealthy suburbs, in garden cities, in quiet country towns. And their children are like unto them.

Alongside feeble folk, dwelling under the same conditions, are healthy folk, big, strong men, muscular, athletic, capable; and brave and hearty women. And when the strong man and the hearty woman mate together their children are like unto them. It is not all immediate environment, it seems.

Their children are like unto them. "Black cats have black kittens." "Feeble stocks give weedy herds to fat pastures." There may be revelations in these commonplace matters. Man, in his own estimation the greatest and most glorious work of the Author of the Universe and the final pinnacle of His glory, forgets his kind is subject to the laws that govern beasts.

